# Segmental chromosomal alterations lead to a higher risk of relapse in infants with *MYCN*-non-amplified localised unresectable/disseminated neuroblastoma (a SIOPEN collaborative study)

**DOI:** 10.1038/bjc.2011.472

**Published:** 2011-11-10

**Authors:** G Schleiermacher, J Michon, A Ribeiro, G Pierron, V Mosseri, H Rubie, C Munzer, J Bénard, N Auger, V Combaret, I Janoueix-Lerosey, A Pearson, D A Tweddle, N Bown, M Gerrard, K Wheeler, R Noguera, E Villamon, A Cañete, V Castel, B Marques, A de Lacerda, G P Tonini, K Mazzocco, R Defferrari, B de Bernardi, A di Cataldo, N van Roy, B Brichard, R Ladenstein, I Ambros, P Ambros, K Beiske, O Delattre, J Couturier

**Affiliations:** 1 INSERM U830, Laboratoire de Génétique et Biologie des Cancers, 26 rue d’Ulm, 75248 Paris Cedex 05, France; 2 Département d’Oncologie Pédiatrique, Institut Curie, 26 rue d’Ulm, 75248 Paris Cedex 05, France; 3 Unité de Génétique Somatique et Cytogénétique, Institut Curie, 26 rue d’Ulm, 75248 Paris Cedex 05, France; 4 Service de Biostatistiques, Institut Curie, 26 rue d’Ulm, 75248 Paris Cedex 05, France; 5 Unité d’Hémato-Oncologie Pédiatrique, Hôpital des Enfants, 31059 Toulouse, France; 6 Département de Biologie et de Pathologie Médicales, Service de Pathologie Moléculaire, Institut Gustave-Roussy, 94800 Villejuif, France; 7 Laboratoire d’Oncologie Moléculaire, Centre Léon-Bérard, 69008 Lyon, France; 8 Children’s Department, Institute of Cancer Research, Royal Marsden Hospital, Sutton SM2 5 NG, UK; 9 Northern Institute for Cancer Research, Newcastle University, Newcastle upon Tyne NE2 4HH, UK; 10 Department of Human Genetics, Newcastle University, Newcastle upon Tyne NE1 3 BZ, UK; 11 Sheffield Children’s Hospital, Sheffield S10 2TH, UK; 12 Paediatric Department of Haematology/Oncology, Children’s Hospital, Oxford OX3 9DU, UK; 13 Department of Pathology, University of Valencia, 46010 Valencia, Spain; 14 Unidad de Oncología Pediátrica, Hospital Infantil La Fe, 46009 Valencia, Spain; 15 Department of Genetics, National Institute of Health Dr Ricardo Jorge, 1649-016 Lisboa, Portugal; 16 Department of Pediatrics, Intituto Português de Oncologia Francisco Gentil, 1099-023 Lisboa, Portugal; 17 Translational Oncopathology, National Cancer Research Institute, 16132 Genova, Italy; 18 Laboratory of Italian Neuroblastoma Foundation, National Cancer Research Institute, 16132 Genova, Italy; 19 Department of Paediatric Haematology and Oncology, Giannina Gaslini Children’s Hospital, 16147 Genova, Italy; 20 Department of Pediatric Hematology and Oncology, University of Catania, 95124 Catania, Italy; 21 Center for Medical Genetics, Ghent University Hospital, 9000 Ghent, Belgium; 22 Cliniques Universitaires Saint-Luc, Université Catholique de Louvain, 1348 Brussels, Belgium; 23 St Anna Children’s Hospital, SIRP-CCRI Studies and Statistics on Integrated Research and Projects, Children’s Cancer Research Institute, Vienna, Austria; 24 CCRI, Children’s Cancer Research Institute, St. Anna Kinderkrebsforschung, 1090 Vienna, Austria; 25 Department of Pathology, Oslo University Hospital, 0424 Oslo, Norway

**Keywords:** neuroblastoma, infants, genomic profile, segmental chromosome alterations, prognosis

## Abstract

**Background:**

In neuroblastoma (NB), the presence of segmental chromosome alterations (SCAs) is associated with a higher risk of relapse.

**Methods:**

In order to analyse the role of SCAs in infants with localised unresectable/disseminated NB without *MYCN* amplification, we have performed an array CGH analysis of tumours from infants enroled in the prospective European INES trials.

**Results:**

Tumour samples from 218 out of 300 enroled patients could be analysed. Segmental chromosome alterations were observed in 11%, 20% and 59% of infants enroled in trials INES99.1 (localised unresectable NB), INES99.2 (stage 4s) and INES99.3 (stage 4) (*P*<0.0001). Progression-free survival was poorer in patients whose tumours harboured SCA, in the whole population and in trials INES99.1 and INES99.2, in the absence of clinical symptoms (log-rank test, *P*=0.0001, *P*=0.04 and *P*=0.0003, respectively). In multivariate analysis, a SCA genomic profile was the strongest predictor of poorer progression-free survival.

**Conclusion:**

In infants with stage 4s *MYCN-*non-amplified NB, a SCA genomic profile identifies patients who will require upfront treatment even in the absence of other clinical indication for therapy, whereas in infants with localised unresectable NB, a genomic profile characterised by the absence of SCA identifies patients in whom treatment reduction might be possible. These findings will be implemented in a future international trial.

In neuroblastoma (NB), the most frequent solid extracranial cancer in childhood, characterised by substantial clinical heterogeneity, several recurrent genetic alterations have been shown to be of prognostic impact (Maris *et al*, 2007; Janoueix-Lerosey *et al*, 2010; Maris, 2010). A near-triploid DNA content, on average reflecting whole chromosome gains, is frequently observed in low-stage tumours of younger children, and is associated with a favourable outcome (Ladenstein *et al*, 2001). On the other hand, *MYCN* amplification and segmental chromosome alterations (SCAs) most often involving chromosome regions 1p, 1q, 2p, 3p, 4p, 11q and 17q are preferentially observed in advanced stages of disease in older children, and are associated with a poorer prognosis. These genetic parameters can now be analysed using genome-wide techniques such as array CGH or SNP arrays, clearly demonstrating that the genetic imbalances combine to define distinct genomic profiles (Vandesompele *et al*, 2005; George *et al*, 2007; Mosse *et al*, 2007; Schleiermacher *et al*, 2007; Tomioka *et al*, 2008; Janoueix-Lerosey *et al*, 2009). Indeed, the presence of SCAs, even in a background of numerical chromosome alterations (NCAs), is associated with a higher risk of relapse and a poorer outcome (Janoueix-Lerosey *et al*, 2009). Although risk stratification schemes have so far integrated molecular data based on only few chromosome loci (Cohn *et al*, 2009), more recent reports suggest that pangenomic data could further improve pre-therapeutic risk estimation (Ambros *et al*, 2009; Janoueix-Lerosey *et al*, 2009; Caren *et al*, 2010). As high-risk NBs nearly always demonstrate SCA, future therapeutic strategies for these cases might rather rely on gene expression or other molecular data (Oberthuer *et al*, 2008; Vermeulen *et al*, 2009; Ambros *et al*, 2011). However, pangenomic data might prove to be especially informative for treatment stratification in the clinically defined low- and intermediate-risk groups (Janoueix-Lerosey *et al*, 2009; Schleiermacher *et al*, 2010). To date, the exact role of genomic imbalances in infants, particularly with localised unresectable/disseminated NB, has not been reported.

The aim of this study was to analyse genetic alterations determined by array CGH in *MYCN*-non-amplified localised unresectable/disseminated NB of infants included in the prospective European INES99.1, INES99.2 and INES99.3 protocols (De Bernardi *et al*, 2009; Rubie *et al*, 2011) and to study the impact of the genomic profile on clinical characteristics and outcome in this population.

## Patients and methods

### Patients

Tumour samples from patients included in the INES99.1, INES99.2 and INES99.3 trials were included in this study (De Bernardi *et al*, 2009; Rubie *et al*, 2011) The INES trials, run by the Société Internationale d’Oncologie Pédiatrique–Europe Neuroblastoma (SIOPEN) in the participating countries Austria, Belgium, France, Italy, Norway, Portugal, Spain, Sweden and United Kingdom, recruited 300 infants aged <12 months diagnosed with a *MYCN*-non-amplified NB from 1999 to 2004. The INES99.1 trial proposed minimal upfront chemotherapy in infants with a localised unresectable NB with the aim to render these tumours resectable (Cecchetto *et al*, 2005; Rubie *et al*, 2011). The INES99.2 and INES99.3 trials proposed chemotherapy for infants with a disseminated NB, in case of life- or organ-threatening symptoms only in stage INSS 4s, or in case of metastases to the bone, lung or CNS (stage INSS 4), respectively (De Bernardi *et al*, 2009). Surgical resection of the primary tumour was performed in the absence of surgical risk factors (Cecchetto *et al*, 2005). In case of disease progression or relapse, individual therapeutic decisions were taken. The protocol was approved by local institutional review boards, and patients were enroled following written informed consent from parents or guardians. Median follow-up of these 300 patients was 60 months; 36 patients have had disease progression or relapse, and 9 patients have died (0 out of 119 in INES99.1, 7 out of 133 in INES99.2 and 2 out of 48 in INES99.3), with a 5-year progression-free survival (PFS) and overall survival (OS) of 87.8% (±1.9) and 97.5% (±0.9), respectively.

### Pangenomic profile

For a total of 218 out of 300 patients, array CGH was performed using DNA extracted from frozen tumour tissue obtained at diagnosis and harbouring >50% tumour cells. Tumours from 93 out of 119 INES99.1, 93 out of 133 INES99.2 and 32 out of 48 INES99.3 patients could be analysed, with array CGH analysis results of 57 samples from French patients having been reported previously (Janoueix-Lerosey *et al*, 2009; Schleiermacher *et al*, 2010). For the remaining patients, either no tumour tissue was available or samples contained an insufficient amount of tumour cells or yielded poor-quality DNA not permitting interpretation of the array CGH result. The absence of *MYCN* amplification was confirmed by fluorescent *in situ* hybridisation in a national SIOPEN reference laboratory and centrally reviewed for all cases (Ambros *et al*, 2009). Among the 218 patients, there have been 30 relapses/progressions, and 5 patients have died.

Following standardised DNA extraction, samples were analysed by array CGH using for 185 cases an in-house BAC/PAC array with a genomic resolution of ∼1 Mb, as reported previously (Janoueix-Lerosey *et al*, 2009; Schleiermacher *et al*, 2010). Using a commercially available NimbleGen DNA array (Roche NimbleGen, Madison, WI, USA), 33 other cases were analysed, containing 72 000 oligonucleotide probes, with an average resolution of ∼1 probe per 40 kb.

All obtained profiles were subjected to detailed visual inspection and analysed using the VAMP software (La Rosa *et al*, 2006). The smoothing algorithm GLAD (Hupe *et al*, 2004) was used to determine the status of the BAC or oligonucleotide probes (normal or altered ratios). A NCA was defined as probe ratios homogeneously altered throughout entire chromosomes, as compared with the median copy number across the genome. A SCA was defined by the presence of either at least 3 contiguous BAC or 100 contiguous oligonucleotide probes exhibiting a genomic status different from that of the rest of the chromosome.

A genomic type was attributed to all analysed samples, taking into account all observed genomic alterations, as described previously, with slight modifications (Janoueix-Lerosey *et al*, 2009; Schleiermacher *et al*, 2010). Cases presenting only NCA, without any SCA, were considered as having a ‘NCA genomic profile’. Cases harbouring SCA, without or with NCA, were considered as having a ‘SCA genomic profile’. Finally, cases in which no genetic changes were observed despite sufficient tumour cell content in the samples were termed ‘silent’ profiles.

The genomic profiles have been deposited in the NCBI Gene Expression Omnibus (Edgar *et al*, 2002) and are accessible through GEO Series accession number GSE26494 (http://www.ncbi.nlm.nih.gov/geo/query/acc.cgi?acc=GSE26494).

### DNA index

The cellular DNA content could be determined by flow or static cytometry in 108 cases. Tumours were classified as di-/tetraploid if the DNA index was ⩽1.2 or ⩾1.8. Tumours with a DNA index between 1.2 and 1.8 were termed pseudotriploid (Ladenstein *et al*, 2001).

### Statistical analysis

Progression-free survival was defined as the time from diagnosis to first event (local or metastatic failure, either during treatment or after completion of treatment) or last follow-up. In patients with INSS stage 4s disease without any specific treatment, progression was taken into account when occurring after 2 months of initial observation. Overall survival was defined as the time from diagnosis to death or last follow-up. Survival curves were analysed according to the Kaplan–Meier method and compared using the log-rank test. Multivariate analysis was performed using the Cox proportional-hazards regression model. Categorical variables were coded as a set of binary ‘yes-no’ variables. A backward model-building procedure was used to identify the variables retained in the Cox model with a *P*-value of ⩽0.05.

## Results

### Pangenomic profiling

**Figure 1:**
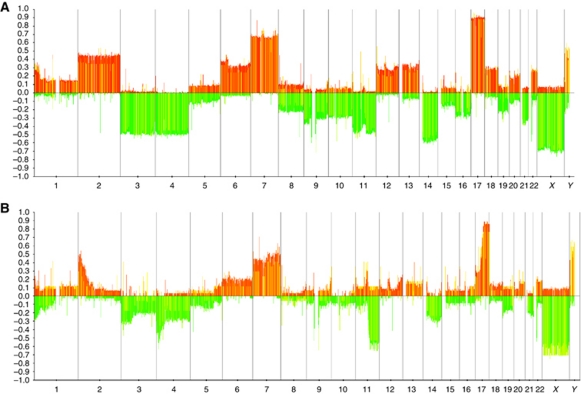
Frequencies of genome copy number gains (*y* axis, positive values, red) and losses (*y* axis, negative values, green) at each locus of the CGH array. Clones are ordered on the *x* axis according to their position in the genome. Vertical grey lines correspond to chromosome separators. (**A**) Cases with a NCA genomic profile (*n*=162): presence of numerical chromosome alterations (NCAs) only. (**B**) Cases with a SCA genomic profile (*n*=48): presence of segmental chromosomal alterations (SCAs) without or with NCA. The colour reproduction of this figure is available at the *British Journal of Cancer* online.

Of the 218 tumour samples analysed by array CGH, no copy number alterations could be detected in 8 cases. Among the remaining 210 cases, a NCA genomic profile was observed in 162 cases, whereas 48 tumours presented a SCA genomic profile (Figure 1). All cases with a SCA genomic profile harboured imbalances of chromosome regions recurrently altered in NB (gain of chromosome arms 1q, 2p or 17q, loss of chromosome arms 1p, 3p, 4p, 11q), except two cases showing imbalances in 14q, and 4q and 6p, respectively.

**Figure 2:**
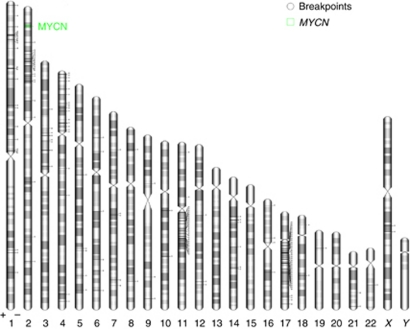
Localisation of breakpoints observed in 48 tumours harbouring SCA. Each breakpoint was localised according to the coordinates of the probe determining the left side of the breakpoint region, according to the Human Genome Draft Hg18 (genome.ucsc.edu/goldenPath/hgTracks.html). The graph was drawn using the web tool Idiographica (http://www.ncrna.org/idiographica/).

Among the 48 cases with a SCA genomic profile, the most frequent SCAs were gain of 17q (81%), gain of 2p (43%), loss of 1p (41%) and loss of 11q (39%) (Figure 1 and Table 1). For most chromosome arms harbouring recurrent imbalances, the breakpoints were scattered over large regions. However, for the 19 tumours harbouring distal 11q loss, breakpoints clustered within a smaller region of 12 Mb (genome position 70–82 Mb; Supplementary Table 1 and Figure 2).

**Figure 3:**
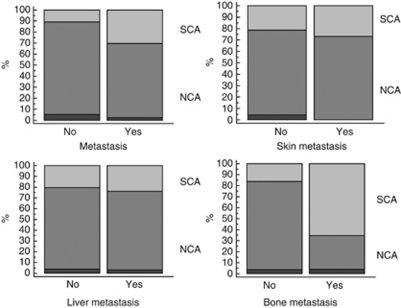
Frequency of genomic types according to the metastatic status (colour codes: dark grey: no genomic alterations; grey: NCA genomic profile; light grey: SCA genomic profile). Here, 38 out of 125 patients with metastases, 7 out of 26 patients with skin metastases and 23 out of 96 patients with liver metastasis had a SCA genomic profile *vs* 10 out of 93 patients without any metastases, 41 out of 192 patients without skin metastases and 25 out of 122 patients without liver metastases (*χ*^2^ test, all NS). Finally, 17 out of 26 patients with radiologically defined bone metastases had a SCA genomic profile *vs* 31 out of 185 patients without bone metastasis (*χ*^2^, *P*<0.0001).

The frequency of the different genomic profiles was analysed in the different study groups, and their distribution in the different groups was not random. Indeed, in INES99.1, 11% of patients had SCA *vs* 20% in INES99.2 and 59% in INES99.3 (*χ*^2^-test, *P*<0.0001; Table 1). In patients with skin, liver or bone marrow metastases, the frequency of SCA was not significantly higher than in patients without such metastases (Figure 3 and Table 1). However, the single chromosome alterations 1p loss, 2p gain, 3p loss, 4p loss, 11q loss and 17q and a SCA genomic profile were observed more frequently in infants with radiologically confirmed bone metastases compared with those without bone lesions (*χ*^2^-test, *P*<0.0001; Table 1).

Among the 108 cases for which ploidy data were available, 33 tumours were diploid/tetraploid and 75 pseudotriploid. The SCA profiles were observed more frequently, but not exclusively, in di/tetraploid tumours (*χ*^2^-test, *P*<0.0001; Table 2).

### Survival analysis

The 5-year PFS and OS for the 218 patients were 86.2% (±2.3) and 97.6% (+/1.0), respectively. Among the 5 patients who died of disease, 1 INES99.2 patient with a NCA genomic profile had bone, bone marrow and liver relapse 14 months after diagnosis, and died of disease 35 months after diagnosis, and 1 INES99.2 neonate with a NCA genomic profile died of fulminant disease shortly after diagnosis. Three other patients (2 INES99.2 and 1 INES99.3) with SCA genomic profiles had bone marrow/liver, bone/bone marrow and bone/skin relapse, and died of disease 10, 10 and 46 months after diagnosis, respectively (Table 3).

**Figure 4:**
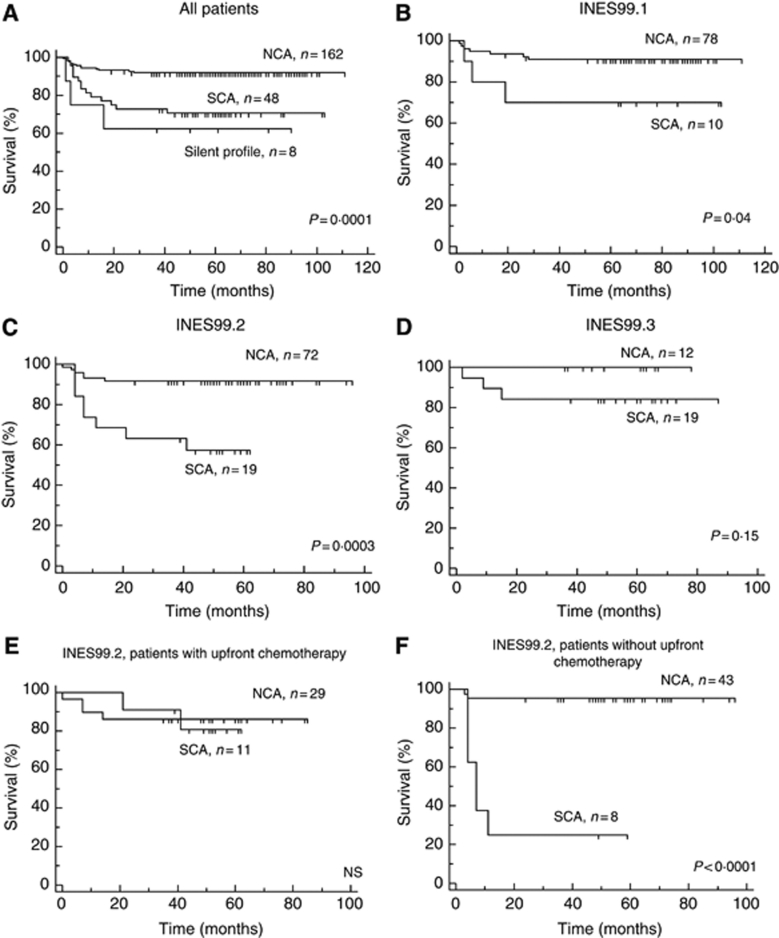
Kaplan–Meier survival analysis showing 5-year progression-free survival (PFS) according to the tumour genomic profile. (**A**) Among the whole study population (*n*=218), the 5-year PFS was 92% (±2.1) in patients with a NCA genomic profile, 70.7% (±6.6) in patients with a SCA genomic profile and 62.5% (±17.1) in patients with a silent genomic profile (log-rank, *P*=0.0001). (**B**) Among infants with a localised unresectable NB (INES99.1), the 5-year PFS was 91% (±3.2) in patients with a NCA genomic profile *vs* 70% (±14.5) in patients with a SCA genomic profile (log-rank, *P*=0.04). (**C**) Among infants with stage 4s NB (INES99.2), the 5-year PFS was 91.7% (±3.2) in patients with a NCA genomic profile *vs* 7% (±11.5) in patients with a SCA genomic profile (log-rank, *P*=0.0003). (**D**) Among infants with stage 4 NB (INES99.3), the 5-year PFS was 100% in patients with a NCA genomic profile *vs* 84.2% (±8.3) in patients with a SCA genomic profile (log-rank, not significant). (**E**) Among infants with stage 4s NB receiving upfront chemotherapy, the 5-year PFS was 86.2% (±6.4) in patients with a NCA genomic profile *vs* 80.8% (±12.2) in patients with a SCA genomic profile (log-rank, NS). (**F**) Among infants with stage 4s NB not receiving upfront chemotherapy, the 5-year PFS was 95.3% (±3.2) in patients with a NCA genomic profile *vs* 25% (±15.3) in patients with a SCA genomic profile (log-rank, *P*<0.0001).

Further survival analyses concerned PFS only. The genetic markers 2p gain, 11q loss and 17q gain were all associated with a poorer PFS (Table 4). A SCA genomic profile was also strongly associated with a poorer PFS (Table 4 and Figure 4). Infants with a SCA genomic profile had a poorer PFS than those with a NCA genomic profile. Interestingly, the eight infants whose tumours had silent genomic profiles also fared worse.

A SCA genomic profile was associated with a poorer PFS in infants with localised unresectable (INES99.1) and with stage 4s NB (INES99.2) (log-rank, *P*=0.04 and *P*=0.0003, respectively). Finally, among infants with stage 4 disease (INES99.3), no statistically significant difference in PFS between patients with a NCA and SCA genomic profile was observed (Figure 4A–D). The DNA index was not of prognostic impact in the studied population.

### Multivariate analysis

To determine which parameters independently predicted PFS, we applied the Cox proportional-hazards procedure including the 210 patients in whose tumours either NCA or SCA profiles had been identified, entering the variables genomic profile, single genetic alterations and treatment group. In a backward model, a SCA genomic profile was found to have a higher risk of relapse (hazard ratio: 5.24, CI 2.4–11.4, *P*<0.0001), whereas a lower risk of relapse was observed for treatment group INES99.3 (hazard ratio: 0.32, CI 0.094–1.11, *P*=0.076). The single genetic alterations were not retained in the model.

### Prognostic impact of genomic profiling in stage 4s patients

In patients with stage 4s disease (trial INES99.2), treatment can be very heterogeneous, with a possibility of observation only, in the absence of clinical symptoms, or necessity of upfront chemotherapy in the presence of clinical symptoms, leading to very different total treatment burdens. We thus sought to analyse the impact of the genomic profile on treatment burden. Among 91 patients with stage 4s disease, 40 patients had symptoms at diagnosis and thus received upfront medical treatment. In this group, no significant difference between PFS of the patients with NCA and typical SCA genomic profiles could be observed. However, for the 51 patients who did not have clinical symptoms at diagnosis and who initially did not receive upfront medical treatment, those with a SCA genomic profile had a significantly lower PFS than those with a NCA genomic profile (Figure 4E and F).

Indeed, for stage 4s patients, both among patients with NCA and with SCA tumours, ∼50% of the patients (43 out of 72 and 8 out of 19 patients, respectively; *χ*^2^ test not significant) did not require upfront chemotherapy, but had medical observation±surgical resection only, indicating that the clinical severity of disease was not worse at diagnosis in the SCA tumour group. However, once having relapsed, only 2 out of 6 (33%) patients with a NCA profile received high-dose chemotherapy for salvage, whereas in the SCA tumour group, 5 out of 8 (62%) patients received such treatment (Table 3).

## Discussion

In NB, different recurrent genetic alterations combine to form distinct genomic types, which are in turn associated with different clinical outcomes. We have recently shown in a large patient cohort that the presence of SCA is associated with a poorer outcome, even when occurring together with NCA, and that tumour progression is frequently associated with an accumulation of SCA, suggesting that SCA could be considered as surrogate markers for an underlying abnormality in a DNA maintenance or repair pathway (Janoueix-Lerosey *et al*, 2009; Schleiermacher *et al*, 2010). We have now explored the hypothesis that in infants with *MYCN-*non-amplified localised unresectable/disseminated NB, pangenomic profiling might provide a useful prognostic marker.

In infants with NB without *MYCN* amplification, overall survival is, fortunately, high. However, in this patient population with a frequent indication for treatment even in newborns, it is crucial to consider treatment burden, as conventional chemotherapy courses, well tolerated in older children, can be associated with significant short- or long-term morbidity. It is thus important to fine-tune treatment indications for these patients in order to avoid over- or under-treatment. Considering that patients of this study have an excellent OS as previously reported (De Bernardi *et al*, 2009; Rubie *et al*, 2011), this study used PFS to determine if pangenomic profiling might be useful for therapeutic stratification in infants with *MYCN-*non-amplified localised unresectable/disseminated NB.

This study confirms recurrent SCA involving chromosome arms 1p, 1q, 2p, 3p, 4p, 11q and 17q in NB, breakpoints in other chromosome arms being much rarer. The data are concordant with previous publications reporting a lower frequency of 1p deletion, 11q deletion or 17q gain in infants with localised NB, and frequencies of 17q gain ranging from 50 to 70% in infants with stage 4s or 4 disease (Spitz *et al*, 2006; Lavarino *et al*, 2009). However, previous studies did not take into account the whole genomic profile. We now show that a genomic profile characterised by the presence of SCA occurs in 11% of infants with localised unresectable NB, 20% of infants with stage 4s and 59% of infants with stage 4 NB. A higher incidence of SCA was observed in older infants, and the median age at diagnosis was higher in infants with a SCA than those with a NCA genomic profile (6.7 *vs* 5.1 months at diagnosis, *t*-test, *P*=0.006).

In infants with disseminated NB, the exact clinical delineation of INSS stage 4s *vs* stage 4 has been controversial (Brodeur *et al*, 1993; Hero *et al*, 2008; Cohn *et al*, 2009; De Bernardi *et al*, 2009). It has been reported that infants with stage 4s disease, including those with a primary tumour crossing the midline, or those with skeletal MIBG uptake in the absence of radiologically proven bone lesions, will require chemotherapy only in the presence of clinical symptoms (De Bernardi *et al*, 2009). We now show that in infants with NB, a SCA genomic profile is associated with metastatic disease and, in particular, bone metastasis. Furthermore, among 185 patients without bone metastasis at diagnosis, 3 out of 154 with a NCA genomic profile progressed with bone lesions *vs* 6 out of 31 with a SCA genomic profile. This observation leads to the hypothesis that the presence of SCA in NB cells might potentially increase their potential to metastasise to bone.

It has been suggested that 11q deletions might be associated with a particularly dismal outcome in older patients (Attiyeh *et al*, 2005; Caren *et al*, 2010). In this study, of the 5 patients who have died of disease, 2 had 11q deletion; and among 17 other patients whose tumours harboured 11q deletion, 5 have relapsed and could be salvaged, indicating that 11q deletion is not associated with a worse OS in infants.

Several recent studies, performed in large patient cohorts encompassing NB patients of all ages, have demonstrated that a genomic profile characterised by SCA is associated with a higher risk of relapse (Tomioka *et al*, 2008; Janoueix-Lerosey *et al*, 2009; Caren *et al*, 2010). We now show specifically in infants with *MYCN-*non-amplified localised unresectable/disseminated NB, in a multivariate setting, that a SCA genomic profile is of prognostic importance, rather than single genetic alterations or clinical stage. No correlation between the size of the individual chromosome alterations and clinical outcome was observed (data not shown). The absence of prognostic impact of the clinical stage in a multivariate setting can probably be attributed to the more intensive chemotherapy for INES99.3 (INSS stage 4) patients, indicating that these patients are sufficiently treated with 4–8 courses of chemotherapy (De Bernardi *et al*, 2009; Baker *et al*, 2010). The type of SCA to be taken into account for the definition of a SCA genomic profile remains controversial, with some recent data supporting the hypothesis that any SCA, whether occurring recurrently or not in NB, may be associated with a poor outcome (Janoueix-Lerosey *et al*, 2009). In this series, only two tumours with a SCA genomic profile harboured only atypical imbalances, precluding from drawing any conclusion on their prognostic impact.

Interestingly, the infants with silent genomic profiles appeared to have a higher risk of relapse. For one sample, a DNA index of 1.46 was noted, indicating a perfect triploid chromosome content, in the context of which NCA might not be detected. On the other hand, it cannot be excluded that smaller alterations not detected by standard resolution arrays might be present in some NBs. The tumours of these patients will merit further exploration using higher-resolution techniques to search for as yet undetected genetic alterations.

Recent studies have enabled the identification of genomic loci associated with NB susceptibility at a constitutional level. These loci have been identified by genome-wide association studies (GWASs), using high-resolution SNP arrays. Common variants within the *FLJ22536*, *BARD1* and *LMO1* genes are significantly associated with susceptibility to high-risk NB, whereas SNPs within *DUSP12*, *DDX4* and *IL31RA* are associated with less aggressive NB (Maris *et al*, 2008; Capasso *et al*, 2009; Diskin *et al*, 2009; Nguyen le *et al*, 2011; Wang *et al*, 2011). Constitutional copy number variations associated with NB susceptibility have been described for *LMO1* at 11p15.4 as well as the *NBPF23* gene at 1q21.1 (Diskin *et al*, 2009; Wang *et al*, 2011). For the former, somatic copy number changes as whole chromosome arm 11p gain were observed in 12% of tumours, but only 5% showed interstitial gain of 11p15. In our study, focusing on genomic profiling of tumour DNA, the lower-resolution array CGH analysis did not detect any copy number alterations of interstitial chromosome regions surrounding the known susceptibility loci 1q21.1 and 11p15.4.

The DNA index was not of prognostic impact in this study using previously published cutoffs. The exact definition of diploid *vs* pseudotriploid tumours based on DNA index remains controversial (Look *et al*, 1984; Ladenstein *et al*, 2001). When using a more restrictive DNA index cutoff of 1 to define diploid tumours, these 11 cases had a poorer PFS compared with the 97 others. However, a SCA genomic profile was associated with a higher risk of relapse even among patients whose tumours had a DNA index of >1 (*P*=0.002).

The prognostic impact of the genomic profile in infants with localised unresectable/disseminated NB without *MYCN* amplification is of high clinical importance. Indeed, the presence of a SCA genomic profile identifies stage 4s NB patients with a higher risk of progression or relapse, for whom salvage therapy might have to be more intense, justifying more upfront treatment in patients who would otherwise receive little or no treatment (De Bernardi *et al*, 2009). On the other hand, a NCA genomic profile defines a population of infants with localised unresectable NB with a lower risk of disease progression. Thus, for infants with localised unresectable NB with a NCA profile, treatment reduction might be possible (Hero *et al*, 2008). These findings will be implemented in a future international trial.

## Figures and Tables

**Table 1:** Frequency of segmental chromosome alterations and genomic profiles according to the study groups and clinical characteristics

					**Liver metastasis**			**Skin metastasis**			**Bone marrow metastasis**			**Bone metastasis**		
**Chromosome alteration**	**INES99.1**	**INES99.2**	**INES99.3**	***P***	**No**	**Yes**	***P***	**No**	**Yes**	***P***	**No**	**Yes**	***P***	**No**	**Yes**	***P***
*Chr 1p*																
Normal	85	81	24	0.006	107	83	NS	168	22	NS	105	85	NS	171	19	0.02
Deletion	3	10	7		10	10		16	4		9	11		14	6	
																
*Chr 2p*																
Normal	85	82	22	0.00006	111	78	0.009	166	23	NS	105	84	NS	171	18	0.006
Gain	3	9	9		6	15		18	3		9	12		14	7	
																
*Chr 3p*																
Normal	88	90	25	<0.0001	112	91	NS	177	26	NS	111	92	NS	183	20	0.003
Deletion	0	1	6		5	2		7	0		3	4		2	5	
																
*Chr 4p*																
Normal	84	87	25	0.02	109	87	NS	171	25	NS	105	91	NS	176	20	0.02
Deletion	4	4	6		8	6		13	1		9	5		9	5	
																
*Chr 11q*																
Normal	85	85	21	<0.0001	105	86	NS	167	24	NS	105	86	NS	175	16	<0.0001
Deletion	3	6	10		12	7		17	2		9	10		10	9	
																
*Chr 17q*																
Normal	81	75	15	<0.0001	97	74	NS	150	21	NS	96	75	NS	160	11	<0.0001
Gain	7	16	16		20	19		34	5		18	21		25	14	

					**Liver metastasis**			**Skin metastasis**			**Bone marrow metastasis**			**Bone metastasis**		
**Genomic profile**	**INES99.1**	**INES99.2**	**INES99.3**		**No**	**Yes**		**No**	**Yes**		**No**	**Yes**		**No**	**Yes**	
NCA genomic profile	78	72	12	<0.0001	92	70	NS	143	19	NS	91	71	NS	154	8	<0.0001
SCA genomic profile	10	19	19		25	23		41	7		23	25		31	17	
‘Silent’ profile	5	2	1		5	3		8	0		5	3		7	1	

Abbreviations: INES=Infant Neuroblastoma European Study; Chr=chromosome; NS**=**not significant; NCA=numerical chromosome alteration; SCA=segmental chromosome alteration.

Cases with a ‘silent’ profile (*n*=8) were excluded from the comparisons for single chromosome alterations.

The data were analysed using the *χ*^2^-test, or Fisher's exact test if necessary.

**Table 2:** Repartition of the genomic profiles according to ploidy

	**Di/tetraploid**	**Pseudotriploid**	***P*-value (** ***χ*** ^**2**^ **)**
Numerical genomic profile	16	65	
Segmental genomic profile	16	8	
Silent profile	1	2	
Total	33	75	*P*<0.0001

**Table 3:** Patients having relapsed, according to the protocol arm and genomic profile

**Protocol**	**Clinical presentation**	**Genomic profile**	**Patient number**	**Site of relapse**	**Time from diagnosis to relapse (months)**	**Treatment after relapse**	**Outcome (FU; months from diagnosis)**
INES99.1		Flat	150	PT	16	Surgery	CR (83)
			165	PT, L, other	1	VP/Carbo × 4	CR (72)
		NCA genomic profile	279	PT, other	13	Surgery	Alive (69)
			100	PT	9	Surgery, VP/Carbo × 2	Alive (59)
			124	PT	15	Surgery	Alive (103)
			63	PT	1	VP/Carbo × 2, CADO × 2, surgery	CR (90)
			26	L	4	Unknown	Alive (52)
			31	Other	33	Chemotherapy, surgery	Alive (96)
			168	PT	26	Surgery, Rx	CR (74)
		SCA genomic profile	227	PT	19	Surgery	Alive (67)
			235	PT	1	VP/Carbo × 2, CADO × 2, surgery	CR (64)
			144	PT, LN	3	VP/Carbo × 2, CADO × 2, surgery	CR (85)
INES99.2	No symptoms at diagnosis; no upfront chemotherapy	Flat	174	PT, L	3	Surgery	CR (49)
		NCA genomic profile	23	PT, S	4	VP/Carbo and CADO (6 courses)	CR (37)
			183	L	3	VP/Carbo × 3	CR (49)
		SCA genomic profile	184	BM, B	7	Topotecan-cyclophosphamide, ICE, HD chemotherapy	CR (49)
			121	B,S	4	VP/Carbo × 2, CADO × 2, VP cyclophosphamide	DOD (10)
			196	L	7	VP/Carbo × 2, CADO × 1	Alive (37)
			14	B, BM	4	VP/Carbo × 2, CADO × 2	Alive (59)
			6	B, BM	11	TVD, HD chemotherapy	Alive (73)
			89	PT, B, BM, S, L, P	4	VP/Carbo, HD chemotherapy, retinoic acid	CR (48)
	Symptoms at diagnosis, requiring upfront chemotherapy	NCA genomic profile	118	B	7	VP/Carbo, HD chemotherapy	CR (36)
			111	B, BM, L	14	VP/Carbo, HD chemotherapy	DOD (35)
			61	L, BM, B, S, LN	7	VP/Carbo × 2, CADO × 2	CR (76)
			172	PT, L	0		DOD (0)
		SCA genomic profile	75	BM, L	41	HD chemotherapy	DOD (48)
			109	B, L, P	21	HD chemotherapy	Alive (40)
INES99.3		SCA genomic profile	81	B	2	Conventional chemotherapy	CR (51)
			185	B, CNS	15	Conventional chemotherapy	Alive (49)
			234	B, BM	9	Conventional chemotherapy	DOD (10)

Abbreviations: INES=Infant Neuroblastoma European Study; PT=primary tumour; S=skin; BM=bone marrow; B=bone; L=liver; P=pulmonary; LN=lymph node; CNS=central nervous system; NCA=numerical chromosome alteration; SCA=segmental chromosome alteration; CR=complete remission; DOD=dead of disease; FU=follow-up; VP/Carbo=etoposide/carboplatin; CADO=cyclophosphamide/vincristin/doxorubicine; TVD=topothecan/vincristin/doxorubicine; ICE=ifosphamide/carboplatin/etoposide; Rx=radiotherapy; HD chemotherapy=high dose chemotherapy with autologous stem cell rescue.

**Table 4:** Prognostic impact of single genetic alterations and genomic profiles

**Marker**	**Status (number of cases)**	**5-year PFS (%±s.e.)**	**Log-rank *P***
Chromosome 1p	Normal (*n*=190)	88.5±2.3	0.09
	Loss (*n*=20)	74.3±9.9	
Chromosome 2p	Normal (*n*=189)	89.4±2.2	0.002
	Gain (*n*=21)	66.3±10.4	
Chromosome 3p	Normal (*n*=203)	86.6±2.4	NS
	Loss (*n*=7)	100	
Chromosome 4p	Normal (*n*=196)	87.7±2.3	NS
	Loss (*n*=14)	78.6±11.0	
Chromosome 11q	Normal (*n*=191)	89.2±2.2	0.0008
	Loss (*n*=19)	63.2±11	
Chromosome 17q	Normal (*n*=171)	91.2±2.4	0.0002
	Gain (*n*=39)	69.0±7.4	
Genomic profile	NCA (*n*=162)	92.0±2.1	0.0001
	SCA (*n*=48)	70.7±6.6	
	Silent (*n*=8)	62.5±17.1	
Ploidy	Di/tetraploid (*n*=33)	83.5±6.8	NS
	Pseudotriploid (*n*=75)	89±3.6	

Abbreviations: PFS=progression-free survival; NS**=**not significant; NCA=numerical chromosome alteration; SCA=segmental chromosome alteration.

For single chromosome alterations, cases with a ‘silent’ profile were not taken into account.
